# Bcl-3: A Double-Edged Sword in Immune Cells and Inflammation

**DOI:** 10.3389/fimmu.2022.847699

**Published:** 2022-03-10

**Authors:** Hui Liu, Lin Zeng, Yang Yang, Chunlei Guo, Hui Wang

**Affiliations:** ^1^ Henan Key Laboratory of Immunology and Targeted Drug, Henan Collaborative Innovation Center of Molecular Diagnosis and Laboratory Medicine, School of Laboratory Medicine, Xinxiang Medical University, Xinxiang, China; ^2^ Department of Translational Medicine Center, The First Affiliated Hospital of Zhengzhou University, Zhengzhou, China

**Keywords:** Bcl-3, NF-κB, immune cells, inflammation, immunity

## Abstract

The NF-κB transcription factor family controls the transcription of many genes and regulates a number of pivotal biological processes. Its activity is regulated by the IκB family of proteins. Bcl-3 is an atypical member of the IκB protein family that regulates the activity of nuclear factor NF-κB. It can promote or inhibit the expression of NF-κB target genes according to the received cell type and stimulation, impacting various cell functions, such as proliferation and differentiation, induction of apoptosis and immune response. Bcl-3 is also regarded as an environment-dependent cell response regulator that has dual roles in the development of B cells and the differentiation, survival and proliferation of Th cells. Moreover, it also showed a contradictory role in inflammation. At present, in addition to the work aimed at studying the molecular mechanism of Bcl-3, an increasing number of studies have focused on the effects of Bcl-3 on inflammation, immunity and malignant tumors *in vivo*. In this review, we focus on the latest progress of Bcl-3 in the regulation of the NF-κB pathway and its extensive physiological role in inflammation and immune cells, which may help to provide new ideas and targets for the early diagnosis or targeted treatment of various inflammatory diseases, immunodeficiency diseases and malignant tumors.

## Introduction

The mammalian nuclear factor kappa B (NF-κB) is a transcription factor of eukaryotic cells and contains several proteins including p65 (RelA), c-Rel, RelB, p50 and p52. These proteins all contain a conserved 300 amino acid N-terminal Rel homology domain that dimerizes these NF-κB subunits to form heterodimers or homodimers, locate in the nucleus, and bind DNA (1). The activation of dimeric NF-κB species is controlled by two independent signaling pathways. One is the classical pathway, which mainly leads to nuclear translocation of dimers containing c-Rel, p65 and/or p50 (2). This pathway is mainly participated in enhancing a valid immune response and helps the regulation of cell proliferation and survival. Another is the non-canonical pathway, it is activated at a slower rate than the classical pathway and is centered on the activation of RelB and p52 subunits, which control essential regulatory genes in body homeostasis (3). Several receptors can activate both pathways and the signaling pathways exist some correlation (4). The inhibitor of NF-κB (IκB) is a family of inhibitory proteins of NF-κB (5). This family includes the typical members IκBα, IκBβ, and IκBϵ, the precursor proteins p105/NF-κB1 and p100/NF-κB2, and the atypical members IκBζ, IκBη, IκBns, and Bcl-3. IκB and NF-κB/Rel protein families can establish a complex interrelationship (6), IκB family ensures feedback regulation of the NF-κB system and can repress or promote transcriptional activity in a stimulus-dependent manner to adapt to the physiological environment.

B-cell lymphoma factor 3 (Bcl-3) is an atypical member of the ikappa B inhibitor (IκB) family (7–10). This gene was first found on *chromosome 19* adjacent to the breakpoints in the translocation *t(14;19)(q32;q13.1)* in some patients with chronic lymphoblastic leukemia (CLL) by cloning and sequencing (10–13). It was first defined as a proto oncogene (11) that contributes to leukemia when it is abnormally expressed. Early study aimed at elucidating the molecular details of Bcl-3. Studies have clarified the structure of Bcl-3 (9). The full-length molecular weight of the Bcl-3 protein is approximately 47 kDa. It contains a proline-rich N-terminal domain, seven central tandem repeat cdc10 domains (i.e., ankyrin repeat domain) and a C-terminal transcriptional activation domain richly containing serine and proline (14, 15). In addition, studies confirmed the binding and regulatory role of Bcl-3 on specific NF-κB dimers (see below).

At present, more and more studies focus on the ability of Bcl-3 can regulate the function of various cells through the NF-κB signaling pathway. In humans, Bcl-3 is also related to the occurrence and development of many diseases and malignant tumors, such as various inflammatory (16–19) and autoimmune diseases (20, 21), blood tumors (22, 23) and solid tumors (24–27). Among them, Bcl-3’s participation in the immune system and various inflammatory diseases have been studied to some extent, and this review will focus on the inflammatory and immune diseases, exploring the current knowledge in this field. Bcl-3 has also been implicated in the occurrence, development, metastasis, invasion and prognosis of various tumors. However, these are not within the scope of this review, and so will not be discussed further.

## Molecular Function and Post-Translational Regulation of Bcl-3

Unlike classical members, Bcl-3 is a nuclear transcription cofactor that is mainly expressed in the nucleus (28). Many studies have explored the regulatory function of Bcl-3 in NF-κB target genes (29–31). Interestingly, Bcl-3 can activate or inhibit NF-κB transcription according to the promoter, cell type or received stimulation (5). Bcl-3 is usually not affected by induced degradation because it contains not only ankyrin repeat domain but also a transactivation domain (11, 15, 32), and it regulates the transcriptional activity of NF-κB target genes by binding to the homologous dimer subunit of p50/p52 (31) or recruit alternative co-modulators (33–35).

**Figure 1:**
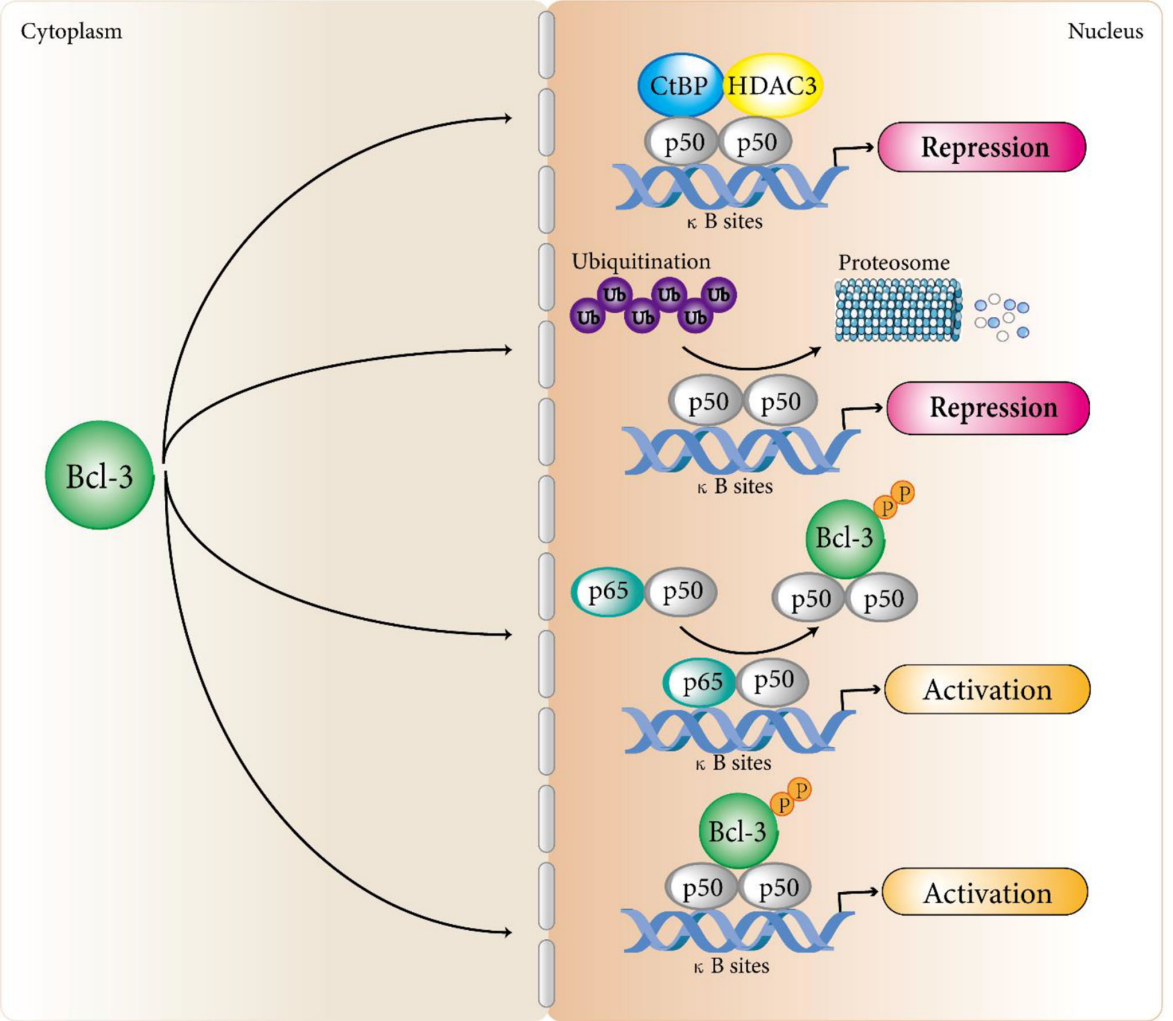
Bcl-3 regulates atypical NF-κB signaling pathways. Bcl-3 acts as a regulator of the atypical NF-κB pathway by binding to processed p50 and p52 homodimers to repress or activate a subset of NF-κB regulated genes. In terms of transcriptional inhibition, Bcl-3 delays the turnover of DNA binding inhibitory p50 homodimer by inhibiting the ubiquitination of p50 homodimer and subsequent proteasome hydrolysis, so as to produce a stable DNA binding complex and inhibit transcription. In addition, the recruitment of co-repressors such as CtBP and HDAC3 may be another mechanism by which Bcl-3 inhibits transcription of NF-κB target genes. In terms of transcriptional activation, Bcl-3 removes repressive p50 homodimers from NF-κB sites, allowing NF-κB heterodimers associated with classical signaling (p65/p50) to activate transcription at these sites. Bcl-3 can also directly transactivate NF-κB-dependent transcription with N-terminal and C-terminal domains by binding to p50 and p52 homodimers. Although Bcl-3 can directly interact with p52 homodimers, the mechanism of Bcl-3 regulating p52 homodimer activation still remains unclear and we speculate that it follows a similar mechanism to that of the p50 homodimers.

Bcl-3 plays a dual function as a regulatory molecule in the transcription of NF-κB (7). The regulation mechanism is as follows:

In terms of transcriptional inhibition, studies by Kerr et al. confirmed that Bcl-3 protein produced in bacteria could suppress the DNA binding activity of some NF-κB members (9). This is consistent with the results of hatada et al. (8) and wulczyn et al. (10). They showed that the human Bcl-3 protein of bacterial expression inhibited the binding of p50 homodimer to DNA. Further exploration found the specific mechanism of Bcl-3 regulating p50 homodimer inhibition. That is, Bcl-3 delays the turnover of DNA binding inhibitory p50 homodimer by inhibiting the ubiquitination of p50 homodimer and subsequent proteasome hydrolysis, so as to produce a stable DNA binding complex and inhibit transcription (36–38). In addition, Keutgens et al. showed that the recruitment of co-repressors such as CtBP and HDAC3 might be another mechanism by which Bcl-3 inhibited transcription of NF-κB target genes (35). In terms of transcriptional activation, Franzoso et al. demonstrated that Bcl-3 could neutralize the inhibitory influence on p50/NF-κB homodimers to help κB site-dependent transcription *in vivo* (30). Further studies revealed a specific mechanism: Bcl-3 removes inhibitory p50 homodimers from NF-κB sites, permitting the connection of NF-κB heterodimers with typical signaling (p65/p50) to promote transcription at these sites (14). On the other hand, Bcl-3 can also directly transactivate NF-κB-dependent transcription with N-terminal and C-terminal domains through binding to 50 and p52 homodimers (9, 32, 39) (**Figure 1**). Similarly, Bcl-3 can also recruit some coactivators to activate the transcription of target genes.

Moreover, Bcl-3 links the p50 and p52 homodimers to the co-regulatory complexes. The function of how these co-regulatory complexes correlate with Bcl-3 is illustrated by the following facts. Dechend et al. proved that Bcl-3 could recruit other co-regulators to affect gene expression. They identified four nuclear cofactors that correlate with the Bcl-3 anchor protein repeat structural domain, namely Pirin, Tip60, Jab1 and Bard1. Among these interacting factors, the histone acetylase Tip60 is able to enhance Bcl-3-p50-activated transcription by NF-κB binding sites, implying that Bcl-3 is an articulator between NF-kB p50/p52 and other transcriptional modulator and activates gene transcription, at least in part, by recruiting Tip60 (33). In contrast, in the study of respiratory syncytial virus, Jamaluddin et al. found that Bcl-3 acted as a virus- derivable depressor of chemokine transcription through influencing the NF-κB and STAT/IRF signaling pathways to decrease the activity of target promoter (40). These studies provide a further dimension to the transcriptional regulation of Bcl-3, with some co-regulators lead to the inhibition of target genes, while others to transactivation. At present, several Bcl-3-interacting proteins have been documented, such as CtBP1/2 (35), β-linked proteins in colorectal cancer cells (41).

The expression of Bcl-3 is strictly controlled. It was confirmed that the Bcl-3 could be modulated by the inducible transcription factors AP-1, STAT3, and NF-κB in various model systems (40, 42, 43). It is worth noting that this dual functional activity of Bcl-3 is controlled by its induced expression and regulated by posttranslational means, including phosphorylation and ubiquitination (38). First, as Bcl-3 is a nuclear coinhibitor, studies have shown that the direct interaction between Bcl-3 and p50 is necessary for stabilizing the p50 homodimer, while Bcl-3-mediated p50 stability is necessary for restricting NF-κB transcriptional activity (37). Phosphorylation of Bcl-3, a nuclear coactivator, is necessary for its transcriptional activity, and nonphosphorylated Bcl-3 is a classical IκB-like inhibitor that can remove p50 and p52 from bound DNA and plays an inhibitory role (15, 31, 44, 45) (**Figure 1**). In many cancer cells, Bcl-3 exists in the form of a phosphorylated protein. Bcl-3 was first found to be extensively phosphorylated in its C-terminal region. Viatour et al. demonstrated that the C-terminal serine residues S361 and S396 of Bcl-3 are targets of GSK3β, which phosphorylates Bcl-3, leading to proteasomal degradation (46). Subsequently, Vivien et al. noted that the transcriptional activity of Bcl-3 requires the phosphorylation of Akt, ERK2 and IKK (47). Akt induced S33 phosphorylation can shift polyubiquitination from K48 to K63, leading to resistance to degradation, translocation to the nucleus and the activation of p52 homodimers. Both S446 phosphorylation by IKK1/2 and S114 phosphorylation by Erk2 can stabilize the BCL3: p52 complex on DNA to strengthen transcriptional activation (47). In addition, some studies have shown that transcriptional activation requires directional ubiquitination in the transactivated region for some DNA-bound transcription factors (48, 49). Ubiquitination of Bcl-3 have important effect on its activation by modulating the intracellular localization of Bcl-3. Inactive Bcl-3 is localized in the cytoplasm in certain cell types, although it primarily located in the nucleus (50, 51), Bcl-3 in the cytoplasm requires K63-linked polyubiquitination for translocation to the nucleus. The de-ubiquitinase CYLD is demonstrated to control the localization of Bcl-3 in keratinocytes by removing these polyubiquitin chains, decreasing the amount Bcl-3 in nuclear and gene transcription mediated by Bcl-3 (52).

In conclusion, Bcl-3 is a strict regulatory protein that regulates the activity of NF-κB. There is evidence that the type of cell, activation stimulus and NF-κB target genes involved determine the regulation and function of Bcl-3. However, how Bcl-3 accommodates NF-κB activity under different physiological or pathological situations is still poorly understood, and elucidating the exact mechanism determining the effect of Bcl-3 is essential for us to understand how the NF-κB family promotes health and disease. Therefore, it still needs exploration.

## Physiological Effects of Bcl-3

Bcl-3 participates in a large number of cellular processes and has a variety of physiological functions as a regulator of NF-κB activity (53, 54). In cells, Bcl-3 can regulate cell proliferation, promote cell survival, participate in the cell cycle and DNA damage response, etc. However, there is evidence that Bcl-3 has different functions and mechanisms in various biological environments and specific cells.

## Bcl-3 Regulates Cell Cycle and Maintains Cell Proliferation

**Figure 2:**
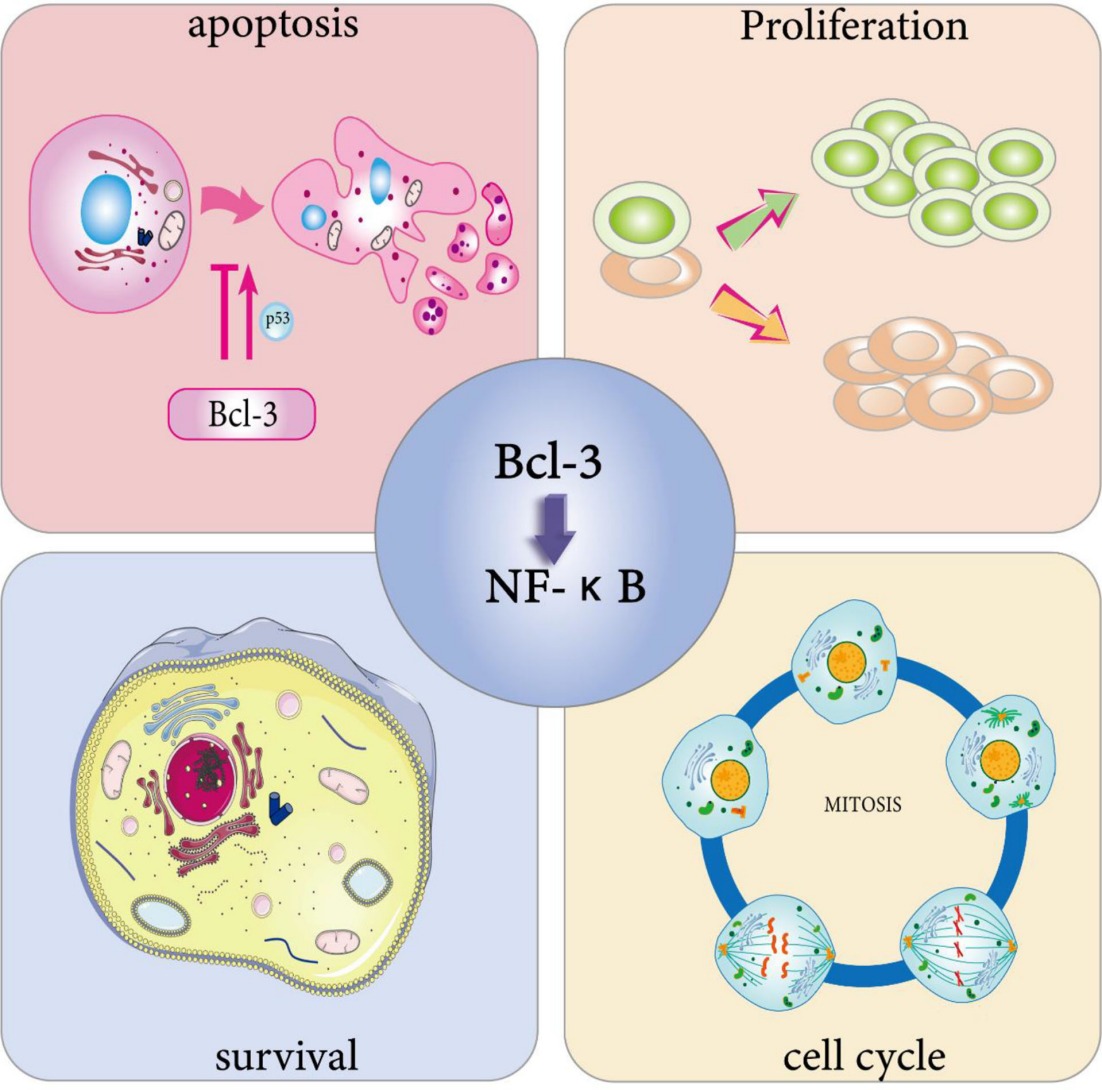
Physiological effects of Bcl-3. Bcl-3 is able to participate in cell cycle regulation. On the one hand, it can directly trigger cell division through the activation of cell cycle protein D, thus promoting cell proliferation, and on the other hand, the overexpression of Bcl-3 slows down T cell proliferation at an early stage during the T cell response to antigen. Bcl-3 has been shown to be a survival gene. In immune cells, activated T cells overexpressing Bcl-3 showed increased survival, while T cells lacking Bcl-3 died abnormally. Bcl-3 has been widely defined as an anti-apoptotic gene. One of its anti-apoptotic pathways involves p53 regulation. However, in survival studies of multiple myeloma (MM) cells, overexpression of Bcl-3 increased apoptosis. The cell characteristics that determine whether Bcl-3 promotes or inhibits the transcription of NF-κB-dependent antiapoptotic genes still need to be identified.

Previous studies have revealed that Bcl-3 expression imbalance enhances the G1 conversion of the cell cycle by provoking the transcription of the cyclin D1 gene in human breast epithelial cells (55). The studies show that Bcl-3 can participate in regulating the cell cycle. Further studies have found the unique mechanism by which p53 regulates the cell cycle process; that is, p53 induces a transcription switch, and the p52/Bcl-3 activation complex is replaced by the p52/HDAC1 repressor complex to actively inhibit the transcription of cyclin D1 (56). However, the study of Feng et al. reached the opposite conclusion (57). When using the human bronchial epithelial cell line BEAS-2B as a model to test the toxic effect of safe doses of arsenite on normal epithelium, they found that low concentrations of sodium arsenite could atypically activate the NF-κB pathway using the human bronchial epithelial cell line BEAS-2B as a model. Cells showed upregulation of p52 and Bcl-3 and nuclear localization, and by passing through the κB site of the cyclin D1 gene promoter, the expression of cyclin D1 was enhanced. The latest study proved that Bcl-3, as a transcription factor that enhances the reprogramming of primordial germ cells (PGCs), significantly enhances the ability of PGCs to reprogram into embryonic germ cells (EGCs) (58). This result reveals that Bcl-3 may enhance PGC reprogramming by controlling the cell cycle (**Figure 2**).

The regulation of cell cycle is one of the reasons why Bcl-3 can induce cell proliferation. It binds to p50 and p52 NF-κB homodimers in the nucleus and activates transcription *via* the transactivation domain of Bcl-3, leading to abundant cell gene expression and cell proliferation (55). In addition to the overexpression of Bcl-3 can activate the transcription of cyclin D1 in human breast epithelial cells (55), it can also act as a coactivator of the AP-1 complex and vitamin AX receptors, both of which can directly trigger cell division by the activation of cyclin D (59, 60), thereby promoting cell proliferation. Zhang et al. found that Bcl-3 also has a positive effect on promoting erythroid proliferation and differentiation (51). In addition, the mRNA and protein levels of Bcl-3 were increased in rats stimulated by thyroid hormone T3 using real-time PCR and Western blot analysis (61). The findings indicate that Bcl-3 may play a key role in T3-induced hepatocyte proliferation (**Figure 2**).

## Bcl-3 Is Involved in Cell Survival and Apoptosis

Bcl-3 has been proven to be a survival gene. In immune cells, activated T cells overexpressing Bcl-3 showed enhanced survival, while T cells lacking Bcl-3 died abnormally (62–64). Other studies have shown that Bcl-3 can mimic the effect of adjuvants; that is, the overexpression of Bcl-3 can not only protect T cells from death but also significantly reduce their activation speed in the early stages of the reaction (65). Similarly, in undifferentiated T cells, upregulation of Bcl-3 by cytokines can prevent activation-induced death of T cells (64). All of the studies above showed that Bcl-3 overexpression leads to increased cell proliferation and survival in a normal physiological environment (**Figure 2**).

In an abnormal physiological environment, Bcl-3 is suggested to facilitate cell survival and proliferation. Studies have shown that cell death is connected with the downregulation of Bcl-3. Bcl-3 expression blocks the apoptosis induced by IL-4 deletion, which suggests that Bcl-3 can act as a survival factor in the absence of growth factors (42). In studying the specific role of Bcl-3 in cells infected with human T cell leukemia virus type 1 (HTLV-1), Gao et al. used shRNA to knock down the expression of Bcl-3. The experimental results revealed that Bcl-3 can promote DNA stability, cell growth and NF-κB activation in HTLV-1-infected cells (66). Moreover, Bcl-3 mutations in lymphocytes give rise to overexpression of IκB protein and uncontrolled cell proliferation, which lead to B-cell chronic lymphocytic leukemia (67). And the recurrent involvement of Bcl-3 in chromosomal translocations in CLL proves that the translocation somehow activates the gene and that its activation is mainly associated with the development of CLL (68). Thus, Bcl-3 may play a role in B-cell survival besides proliferation.

Due to the survival advantage provided by the abnormal expression of Bcl-3 in cells, there is no doubt that it will have a certain impact on apoptosis. On the one hand, Bcl-3 is widely considered an anti-apoptotic gene (**Figure 2**). Studies have shown that Bcl-3 plays a key role in UVB-induced apoptosis. Mitochondrial (endogenous) and death receptor (exogenous) apoptosis pathways are activated in Bcl-3-silenced cells that receive UVB radiation, implying that Bcl-3 inhibits apoptosis after UVB radiation exposure by modulating two apoptotic pathways. Moreover, with the increase in the DNA repair protein DNAPK content, the antiapoptotic function of Bcl-3 may be correlated with DNA damage (69). In addition to the above studies, other studies have shown that Bcl-3 contributes to the regulation of p53; that is, HDM2 dependent on Bcl-3 expression inhibits p53-induced apoptosis (70). This study shows that the abnormal expression of Bcl-3 may not only promote the expression of Hdm2 to prevent DNA damage-induced apoptosis but also inhibit p53-mediated target gene-induced apoptosis. In addition, the antiapoptotic activity of Bcl-3 was displayed in T cells, and it inhibited the proapoptotic activity of Bim (64).

However, on the other hand, Richard et al. noted that enhanced Bcl-3 expression was not the reason for the antiapoptotic effect of IL-9 against glucocorticoid-induced T-cell lymphoma apoptosis (71). Meanwhile, overexpression of Bcl-3 increased cell apoptosis, although Bcl-3-specific siRNA did not affect the viability of a multiple myeloma (mm) cell line (ina-6) (72). Therefore, the above studies show that the cell characteristics that determine whether Bcl-3 promotes or inhibits the transcription of NF-κB-dependent antiapoptotic genes still need to be identified.

## Bcl-3 and Inflammation

## Bcl-3 as an Anti-Inflammatory Factor

**Figure 3:**
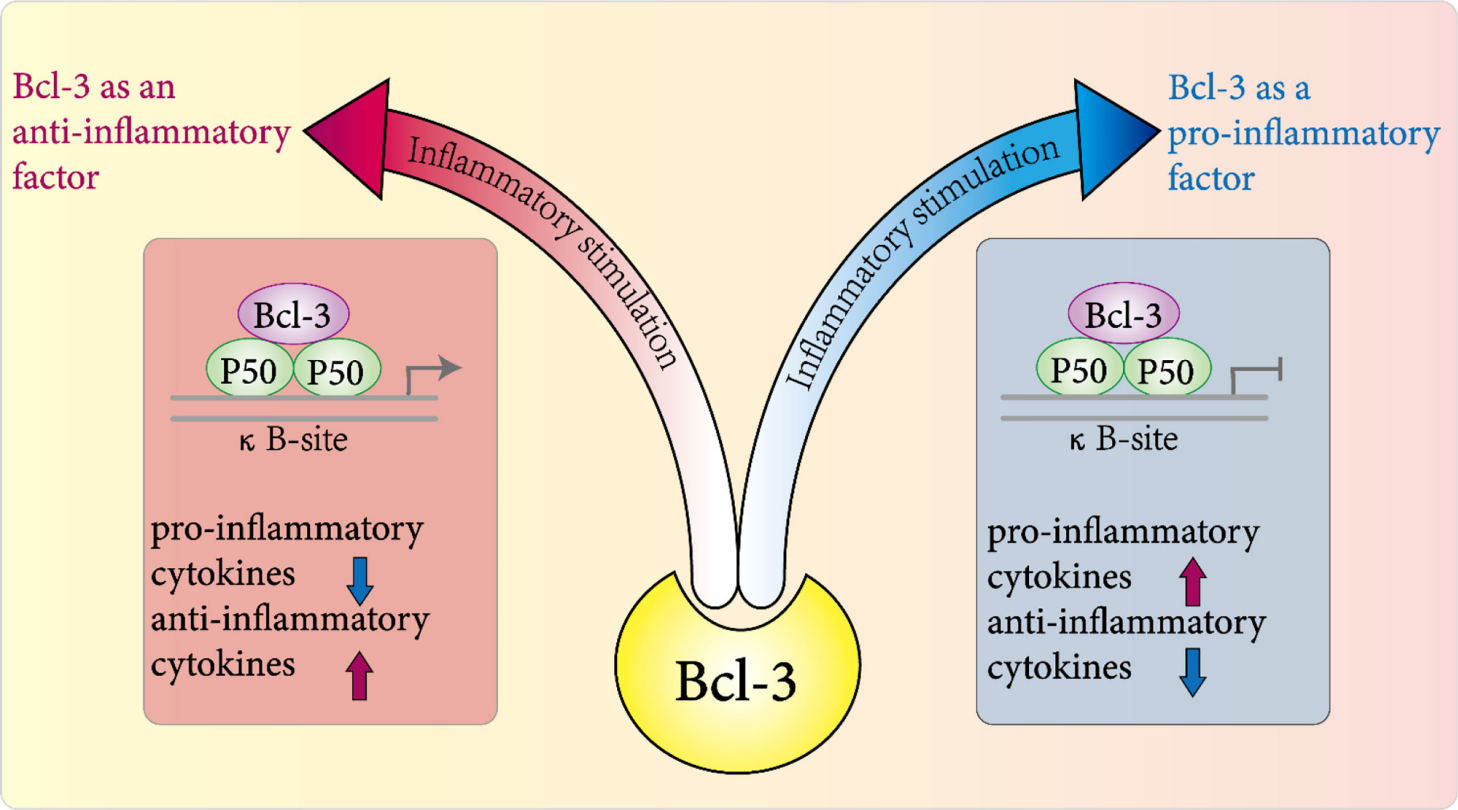
Role of Bcl-3 in inflammation. In various disease models, it plays a pro-inflammatory or anti-inflammatory role directly or indirectly in the host's inflammatory response to pathogens by regulating the expression and release of a variety of inflammatory factors.

NF-κB activity is regulated by a complex mechanism including various proteins, and it plays a key role in the initiation and resolution of inflammation (73–75). Therefore, the NF-kB modulator Bcl-3 is important in the host’s defense against certain pathogens (54). In nearly two decades of research, Bcl-3 has been widely regarded as an anti-inflammatory factor that regulates the expression of NF-κB target genes in different inflammation models (**Figure 3**). In the inflammatory response against pathogen invasion and infection, for example, in the Gram-negative Klebsiella pneumonia model, Bcl-3 regulates the production of cytokines, especially by promoting the expression of IFN-γ and inhibiting IL-10 to indirectly control inflammation (76). A study by Kuwata et al. also clarified that the mechanism by which IL-10 inhibits the production of cytokines by macrophages is related to Bcl-3; that is, Bcl-3 inhibits LPS-induced production of TNF-α by binding to NF-κBp50 (77). Moreover, Bcl-3 may participate in the recruitment of chemokines for neutrophils and help neutrophils kill bacteria (76). In summary, these results demonstrated that Bcl-3 indirectly controls the coordinated response to bacterial attack by regulating the production and release of cytokines in multiple cell types.

In addition to the inflammatory response caused by microbial infection, Bcl-3 plays a key role in the absence of microbial inflammation (sterile inflammation) (78). Bcl-3 expression was upregulated during the control of aseptic inflammation of the mouse pancreas and biliary system. Bcl-3 may decrease the inflammatory response in these tissues by preventing ubiquitination and proteasome-mediated degradation of p50 homodimers, thereby prolonging the binding between NF-κB heterodimers and DNA. It is worth noting that this anti-inflammatory role of Bcl-3 may be the main function of Bcl-3 in aseptic inflammation or pathogen-induced inflammation.

In addition to the above inflammatory models, a similar role of Bcl-3 was reported in other inflammatory systems. To identify potential therapeutic targets for acute renal injury (AKI), Poveda discussed the expression of Bcl-3 in renal tubular cells and the regulation of Bcl-3 function (79). Bcl-3 was the most upregulated NF-κB-related gene in experimental acute kidney injury, and it was also one of the most upregulated genes in renal tubular cells stimulated by TWEAK. Further studies have found that Bcl-3 is a key modulator of NF-κB during kidney injury from inflammatory stimuli (such as TWEAK). In renal cells, inflammatory stimulation prompts the expression of Bcl-3, which in turn directly helps to prevent the inflammatory and lethal responses of renal tubular cells induced by inflammatory stimulation and other cytokines. In addition, Bcl-3 limits granulocyte production under inflammatory conditions, thus preventing acute inflammatory lung injury in mice (80). Coincidentally, the same studies have noted that decreased Bcl-3 expression is associated with a high risk of multiple sclerosis (81).

Moreover, Bcl-3 is indispensable in the process of dendritic cells initiating or activating the adaptive T cell immune response to *Toxoplasma gondii* (82). Bcl-3 was reported to play a vital role in the late stage of the contact hypersensitivity (CHS) response. It can indirectly limit inflammation by resisting the long-term generation of chemokines and other mediators by radiation cells (including keratinocytes) (83). In the development of autoimmune diseases, Bcl-3 has also been shown to play an anti-inflammatory role. For example, in the phenotype of Bcl-3 deficient BL6/LPR mice, Tang et al. revealed that Bcl-3 relies on TNF to inhibit the lupus phenotype of BL6/LPR mice (84), and Bcl-3 deficiency in hematopoietic cells can also directly increase susceptibility to diabetes (85). Mufazalov et al. found that the overexpression of T cell-specific Bcl-3 led to the reduction of intestinal inflammation in mice with anti-CD3 antibody, and the impaired development of Th17 cells led to the resistance of mice with Bcl-3 overexpression to EAE (86).

## Bcl-3 as a Proinflammatory Factor

On the other hand, an increasing number of studies have found that Bcl-3 also plays a role as a proinflammatory factor in some inflammatory diseases (**Figure 3**). In earlier studies, Kazuyuki et al. found that the signal intensity of Bcl-3 in CD4^+^ T cells of patients with rheumatoid arthritis was positively correlated with the signal intensity of the Tfh cell-related genes CXCR5, inducible costimulatory factor (ICOS) and ASCL-2 (87). These findings show that Bcl-3 participates in the development of Tfh cells by inducing the production of IL-21, thus indirectly participating in the pathogenesis of rheumatoid arthritis and acting as a proinflammatory factor. In addition, Nadine et al. revealed that in nonalcoholic steatohepatitis (NASH), using a transgenic mouse model with hepatocyte specific Bcl-3 overexpression, hepatocyte-specific Bcl-3 directly accelerates hepatic steatosis and inflammation *via* insulin-sensitive metabolic transcription factors (88). Bcl-3 was reported to have proinflammatory effects in different disease models.

In T cell-dependent autoimmune diseases, studies have found that deletion of Bcl-3 in T cells blocks colitis and experimental autoimmune encephalomyelitis caused by T cell metastasis (89). Further studies found that the protection of disease correlated with the production of the cytokine IFN-γ, which is related to the decrease in Th1 cells in GM-CSF and the increase in Th17 cells. This indicates that Bcl-3 is important for the pathogenicity of CD4^+^ T cells and the stability of Th1 cells. In chronic inflammatory diseases of the digestive tract, O’carroll et al. used the colitis model of *Bcl-3* ^-/-^ mice induced by dextran sodium sulfate (DSS) and found that compared with the wild-type control, *Bcl-3* ^-/-^ mice were less sensitive to DSS induced colitis and there was no significant difference in the level of proinflammatory cytokines. In addition, intestinal epithelial cell proliferation analysis revealed enhanced proliferation of small intestinal epithelial cells in *Bcl-3* ^-/-^ mice (90). This is completely contrary to the conclusion of Fransen et al. that the expression of the Bcl-3 gene is reduced as a potential risk factor for Crohn’s disease through a single-nucleotide polymorphism analysis (SNP) (91). These above studies suggest that the role of Bcl-3 in the intestinal epithelium is an important regulator of mucosal inflammation. Recently, Sonja et al. showed that Bcl-3 has a similar protective role in mucosal inflammation. They noted that the overexpression of T cell-specific Bcl-3 causes a decrease in NF-κB activity, which gives rise to abnormal development and function of Tregs, causing spontaneous colitis (92). Thus, we clarify that overexpression of Bcl-3 affects T-cell function, whereas Bcl-3 deficiency acts by enhancing epithelial cell survival, targeting the activity of Bcl-3 in inflammatory bowel disease may be a valid tactic to inhibit intestinal inflammation.

In conclusion, these studies show that the role of Bcl-3 in inflammation may not be unilateral. It can not only inhibit the occurrence and development of inflammation as an anti-inflammatory factor but also participate in the process of inflammation as a pro-inflammatory factor, although its specific regulatory role is not clear. However, targeting Bcl-3 activity will undoubtedly become a potential pathway and emerging treatment target for various inflammatory diseases.

## Mechanisms of Bcl-3 Related Inflammation

In studies on the mechanism of the correlation between Bcl-3 and inflammation, the proinflammatory properties of Bcl-3 are mentioned. The latest study defines the relevant mechanism by which an increase in Bcl-3 levels restricts the development and function of Treg cells, leading to spontaneous colitis (92). That is, Bcl-3 and NF-κB are directly associated with p50 to inhibit p50/p50, and the p50/p65 NF-κB dimer binds to DNA in Treg cells, thus modulating NF-κB-mediated gene expression. An increase in the Bcl-3 expression level will lead to a decrease in NF-κB activity, which will lead to abnormal Treg development and function and cause spontaneous colitis.

Interestingly, referring to the anti-inflammatory mechanism of Bcl-3, studies have reported that NF-κB binding motifs pinpoint Toll-like receptor-induced gene suppression by inducible blockers (93). In addition, the specificity of the innate immune response is achieved by NF-κB p50 inhibition of interferon response elements (94). These two studies highlight the significance of p50 in modulating transcriptional programs during inflammation. This view was confirmed by Collins et al, who used fixed peptide array technology to report for the first time that NF-κBp50 is a vital target of Bcl-3 anti-inflammatory activity. By defining the p50 region needed for the generation of the Bcl-3 p50 homodimer immunosuppressant complex, they demonstrated that the direct interaction between amino acids 359-361 and 363 of Bcl-3 and p50 is necessary for stabilization of the p50 homodimer and the anti-inflammatory function of Bcl-3 (37). For further research, Collins et al. designed a Bcl-3 mimic peptide based on the ANK1 region of Bcl-3 (95). In the carrageenan-induced foot swelling mouse model, the mimic peptide of Bcl-3 was proven to be valid in suppressing inflammation *in vivo*. In conclusion, through the above studies, the direct interaction of p50 and Bcl-3 was successfully proven to be necessary for Bcl-3-mediated restriction of proinflammatory gene expression.

In addition, previous studies demonstrated that Bcl-3 is an important negative regulator of NF-κB in the signal transduction process of Toll-like receptors and TNF receptors (38). Its regulatory mechanism is as follows: Bcl-3 restrains the ubiquitination of the p50 homodimer and subsequent proteasome degradation, thereby limiting the expression of proinflammatory factors after activation of Toll-like receptors and limiting the force of the TLR response and maintaining natural immune homeostasis. These results show that harmful inflammatory diseases can be regulated by selectively targeting the p50 ubiquitination pathway. Studies have confirmed the view that NF-κBp50 and Bcl-3 play an anti-inflammatory regulatory role in LPS-induced macrophage inflammation by inhibiting the transcription of proinflammatory cytokines and activating the expression of IL-10 (96). However, Bcl-3 limits TLR-induced MAPK activity by modulating the stability of Tpl-2 kinase and determines the level of TLR ligands needed to trigger the inflammatory response (97). Finally, Song et al. also showed that the upregulation of Bcl-3 expression in the aseptic inflammation of mouse pancreas or biliary tract tissues appears to extend the NF-κB heterodimer binding to DNA to reduce inflammation in these tissues by blocking ubiquitination and proteasome-mediated degradation of the p50 homodimer (78).

Recent studies suggest that Bcl-3 may be a new metabolic regulation factor that regulates lipid metabolism during the development of obesity (98). The level of Bcl-3 was highly expressed in the adipose tissue of obese mice. Therefore, the obesity model of Bcl-3^-/-^mice was established by a high-fat diet for 16 weeks. They considered that Bcl-3 to be a potential modulator of fatty acid synthesis, and it can further affect the lipid metabolism of liver and adipose tissue by downregulating the expression of SREBP1, Fas and ACC as well as reduce inflammation in fat and liver tissue.

In conclusion, NF-κB is important in inflammation (74) and cell survival, and Bcl-3 can regulate NF-κB-regulated transcription in a context-dependent manner. Thus, we conclude that highly context-dependent effects of Bcl-3 in the progress of inflammation-associated pathology. For example, in macrophages, Bcl-3 was identified as an inducible gene for the anti-inflammatory factor IL-10. IL-10 induced by Bcl-3 negatively regulates the LPS-induced production of TNF-α (77). However, in alveolar macrophages, after stimulation with LPS or heat-inactivated Klebsiella pneumoniae *in vitro*, alveolar macrophages lacking Bcl-3 had higher levels of IL-10 and an almost complete absence of IFN-γ (76). Not coincidentally, in T-cell-dependent autoimmune diseases, deletion of T-cell-specific Bcl-3 exacerbated part of the lupus-like phenotype (84) but blocked T-cell transfer resulting in colitis and experimental autoimmune encephalomyelitis (89). In conclusion, in various disease models, it plays a pro-inflammatory or anti-inflammatory role directly or indirectly in the host’s inflammatory response to pathogens by regulating the expression and release of a variety of inflammatory factors (54).

## Bcl-3 as a Bidirectional Regulator in Immune Cells

**Figure 4:**
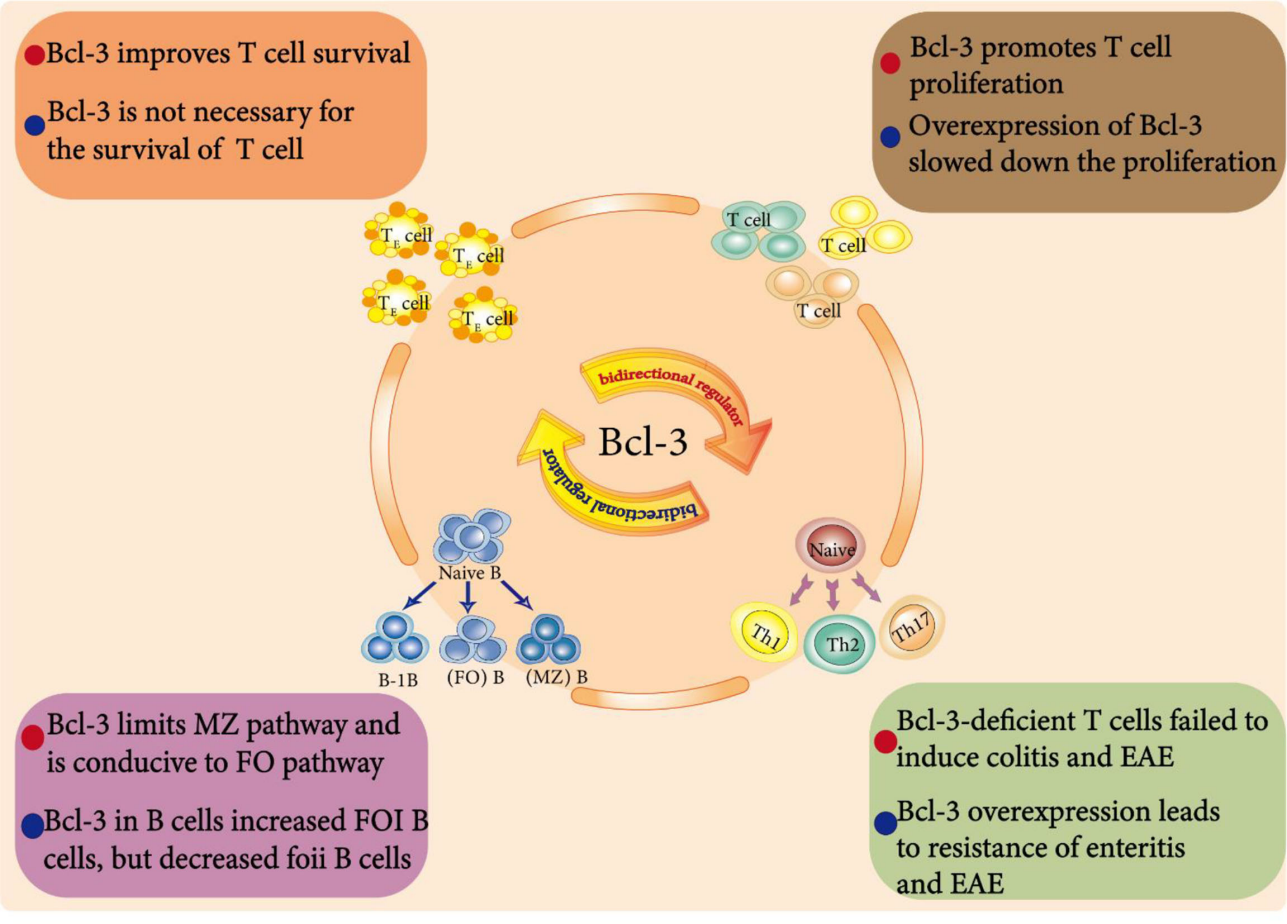
Bcl-3 as a bidirectional regulator in immune cells. Endogenous Bcl-3 is crucial for the development and function of B cells. For example, Bcl-3 regulates the development of splenic B cells. It limits the development of marginal zone (MZ) B cells but is conducive to the development of follicular (FO) B cells. In addition, Bcl-3 is an environment-dependent T cell response regulator that affects T cell survival, differentiation and proliferation and plays a dual regulatory role in them.

Members of the NF-κB transcription factor family are important regulators of the immune response and control the expression of many immune regulatory genes (99). The regulatory ability of Bcl-3 on specific NF-κB dimers has been intensively studied, but its physiological function *in vivo* has still not been comprehensively investigated. Bcl-3 is regarded as an environment-dependent immune cell response regulator (100) that can influence different immune cells and have dual effects due to the cell type, signal and genes (**Figure 4**).

## Bcl-3 in B Cell Development

Naive B cells can be divided into B-1B cells, follicular (FO) B cells and marginal zone (MZ) B cells. Zhang et al. showed that Bcl-3 regulates the development of splenic B cells; that is, Bcl-3 limits the development of marginal zone (MZ) B cells but is conducive to the development of follicular (FO) B cells (101).

Bcl-3 promoted the survival of BCR-stimulated cells, thus promoting the overall proliferation of cells, but it weakened the response to LPS. These results show that Bcl-3, as a regulator determining the fate of B cells, can limit the MZ pathway and favor the FO pathway by increasing signal-specific survival, which is related to its tumorigenic activity. To further study the effect of Bcl-3 on B cell maturation and regulation, Hövelmeyer and others cultivated Bcl-3 mice specifically overexpressing B cells. Notably, the number of FOI B cells in these mice was increased, and the number of FOII B cells was decreased. In addition, the proliferation of B cells in these mice was also impaired (102).

Overall, endogenous Bcl-3 is crucial for the development and function of B cells, and Bcl-3 may play different roles according to different cells, signals and genes. However, the exact molecular mechanisms are not completely clear. Therefore, further investigation is required to clarify the specific function of Bcl-3 in the adaptive immune cell population.

## Bcl-3 in T Cell Survival, Differentiation and Proliferation

Previous data have shown that adjuvants can prolong the life expectancy of activated T cells as well as the overexpression of Bcl-3. Mitchell et al. found that Bcl-3 can promote survival and that T cells activated by two different adjuvants upregulated the expression of the Bcl-3 gene through a microarray analysis (63). Further retroviral infection experiments also showed that a short domain in Bcl-3 is related to its lymphocyte survival activity and that Bcl-3 could improve the survival rate of activated T cells both *in vitro* and *in vivo* (103). However, Bauer et al. reported that the cytokines of adjuvant-triggered DCs induced the overexpression of Bcl-3 and increased the survival rate, but the survival induced by cytokines was not completely dependent on Bcl-3. Bcl-3 controlled the death of activated T cells only by regulating the BH3 proteins Bim and puma (64). Recent studies have also proposed that the high expression of Bcl-3 promotes the survival of CD4^+^ T cells (86). Chilton et al. found that Bcl-3 is necessary for CD8^+^ T cells to continuously produce IFN-γ and can produce the maximum amount of IFN-γ after secondary exposure to antigen, but it is not necessary for the survival effect induced by adjuvant (104)

Moreover, Bcl-3 was reported to participate in the generation of differentiated CD4^+^ Th cells. Bcl-3 can promote Th2 cell differentiation by activating GATA3 through transcription (105). Loss of Bcl-3 in T cells may not affect the differentiation of Th1 cells, differentiated Th1 cells are transformed into low pathogenic Th17-like cells (89). while Mufazalov et al. reached a completely opposite conclusion, they found that overexpression of T cell-specific Bcl-3 led to impaired development of Th1, Th2 and Th17 cells. Although the high expression of Bcl-3 promoted the survival of CD4^+^ T cells, it also inhibited proliferation in response to TCR stimulation, leading to a reduction in the expansion of CD4^+^ T cell (86).

In addition, Rangelova et al. found that the T cells of FADD-DN/Bcl-3^-/-^ mice displayed severe proliferation inhibition and a large amount of cell death upon stimulation with TCR *in vitro (* 62). While Bassetti et al. confirmed the inhibitory effect of Bcl-3 on proliferation and found that whether *in vitro* or *in vivo*, Bcl-3 overexpression slowed the proliferation of T cells in the early stage of the T cell response to antigens. This characteristic is inherent to T cells overexpressing Bcl-3 and does not require the assistance of other cells, such as antigen-presenting cells (65).

In conclusion, these studies show that Bcl-3 is an environment-dependent T cell response regulator that can influence the survival, development and proliferation of T cells. However, the molecular mechanisms supporting cell functions remain to be uncovered, and it will be interesting how research in this field will develop in the future.

## Prospect

In conclusion, Bcl-3 can promote or inhibit the expression of NF-κB target genes according to the cell type and the stimuli received, thereby playing an important role in a variety of physiological functions in the cell. In cells, Bcl-3 can regulate cell differentiation, control cell proliferation, promote apoptosis, and participate in the cell cycle and DNA damage response. In the human body, Bcl-3 is also closely related to various immune and inflammatory pathologies and is related to the occurrence, development, invasion and prognosis of various blood and solid tumors.

In terms of the inflammatory response, Bcl-3 can regulate the mouse inflammatory response by regulating the expression and release of a variety of inflammatory factors. However, whether Bcl-3 is an anti-inflammatory factor or a pro-inflammatory factor has not been clarified. In addition, its anti/proinflammatory mechanism in the specific pathological environment is not very clear.

In terms of immune function, upregulated or downregulated Bcl-3 protein expression will have a negative impact on some effects of immune function. Therefore, the balance of Bcl-3 protein expression may be key to maintaining the normal operation of the human adaptive immune response. The function and potential mechanism of Bcl-3 in specific immune cells still need to be further studied.

Over the past two decades, metabolic changes have been increasingly recognized to be the basis for immune cells to perform certain functions. Meanwhile, a new concept of shaping innate or acquired immune responses through the manipulation of cell metabolism has been proposed. Interestingly, studies have revealed that the ability of dendritic cells to initiate T cells is related to the significant transformation of metabolic programming from oxidative phosphorylation to aerobic glycolysis (106), and similar processes also occur in highly proliferative and stressed tumor cells and activated lymphocytes. Moreover, Bcl-3 can regulate lipid metabolism in the occurrence and development of obesity (98), which implies that the regulation of Bcl-3 on inflammation and immune cells may be related to metabolism and indicates an emerging mechanism by which Bcl-3 regulates cell function through metabolism.

## Author Contributions

HL and LZ conceived and designed the project. HW supervised this project. YY and CG conducted edited the manuscript. HL wrote the manuscript, and all authors discussed the results and proofread this paper.

## Funding

This work is supported by the National Natural Science Foundation of China (81901590, 81871309).

## Conflict of Interest

The authors declare that the research was conducted in the absence of any commercial or financial relationships that could be construed as a potential conflict of interest.

## Publisher’s Note

All claims expressed in this article are solely those of the authors and do not necessarily represent those of their affiliated organizations, or those of the publisher, the editors and the reviewers. Any product that may be evaluated in this article, or claim that may be made by its manufacturer, is not guaranteed or endorsed by the publisher.
